# Dynamic characteristics and reinforcement mechanism of silty soil improved by regenerated fiber polymer

**DOI:** 10.1038/s41598-023-45281-2

**Published:** 2023-10-25

**Authors:** Lulu Liu, Jinpeng Zhao, Xiaoyan Liu, Shixin Lv

**Affiliations:** 1 State Key Laboratory for Geomechanics and Deep Underground Engineering, China University of Mining and Technology, Xuzhou, 221116 China; 2 Institute of Geotechnical Engineering, School of Transportation, Southeast University, Nanjing, 211189 China; 3 School of Highway, Chang’an University, Xi’an, 710064 China; 4 School of Mechanics and Civil Engineering, China University of Mining and Technology, Xuzhou, 221116 China

**Keywords:** Civil engineering, Sustainability

## Abstract

The development of economical and efficient roadbed silt reinforcement technology not only guides the proficient utilization of silt resources but also fosters the green development of geotechnical engineering construction. Ensuring the robust dynamic stability of the roadbed is pivotal for the safe operation of vehicular traffic. In this context, employing recycled polyester fibers and inorganic curing agents, this paper leverages dynamic triaxial testing and scanning electron microscopy (SEM) methods to scrutinize the dynamic characteristics and microscopic mechanisms of recycled fiber polymer modified roadbed silt. The findings indicate that: (1) with a fiber content of 0.2%, the fiber-modified soil sample exhibits minimal damage, maximal dynamic strength and dynamic elastic modulus, and optimum resilience to dynamic loads; (2) akin to fiber-reinforced sand, an elevation in confining pressure can induce the creation of a quasi-cohesive force in fiber-reinforced soil, equivalently enhancing the confining pressure and thereby amplifying its strength; and (3) the surfaces of microparticles in the enhanced soil structure are enveloped with cementitious substances, while smaller soil particles coalesce to form aggregates that fill inter-particle pores, cultivating a denser and more stable improved soil structure and augmenting the dynamic characteristics of the improvement investment.

## Introduction

In the realm of traffic engineering in coastal and riverside locations, challenges frequently arise in the treatment of silty soil subgrades. Such subgrades are predisposed to dust emission during dry construction and are susceptible to collapse when water-saturated. Moreover, they exhibit difficulties in compaction and demonstrate suboptimal performance as subgrade fill materials^1,2^. In real-world projects, high-quality fillers for subgrades are often scarce, leading to significant increases in overall project costs when silty soils are replaced. Thus, there is an imperative to identify cost-effective and efficient technologies for improving the characteristics of silty soil subgrades^3^.

Soil reinforcement technology, particularly for silty soil, represents a focal point and challenge in the domain of international geotechnical engineering^4,5^. Current advancements have shown that the engineering properties of silty soil can be markedly enhanced through the incorporation of inorganic materials such as cement, lime-ash, and lime. Post-reinforcement, the soil exhibits substantial improvements in all engineering performance metrics. Due to its myriad benefits, soil reinforcement techniques have found extensive applications in road engineering, yielding notable social and economic advantages^6^. Several researchers have also substantiated these claims. For instance, Xue et al.^7^ determined an optimal cement mixing ratio of 18% for reinforced silty soil based on indoor tests. Naeini and Baziar^8^ conducted cyclic triaxial tests on geotextile-reinforced silt, examining both reinforced and unreinforced conditions, as well as varying silt content. Yang and Yu^9^ investigated the dynamic behavior of reinforced silty sand using consolidated-undrained dynamic triaxial tests, noting an increased dynamic elastic modulus with reinforcement and elevated confining pressure or consolidation stress ratio. Zhang et al.^10^ analyzed displacement characteristics of ground surfaces and tunnels beneath silty foundations using field monitoring tests and numerical simulations, suggesting potential reinforcement methods such as silt dewatering and vertical jet grouting. Despite its merits, reinforced soil is susceptible to brittle failure, which can lead to pavement cracking^11^. Moreover, existing techniques have certain drawbacks in the realm of environmental sustainability^12,13^. Consequently, there is an urgent need to develop new, efficient, and environmentally sustainable materials for silty soil reinforcement.

Fiber reinforcement technology represents a contemporary advancement in the field of soil engineering, aiming to bolster various soil performance metrics by uniformly integrating scattered fiber filaments into the soil matrix^14,15^. This approach has demonstrated considerable efficacy in a range of soil types, including expansive, soft, lime, cement, and saline soils. It has been shown to significantly enhance soil shear strength, tensile strength, compressive strength, and bearing capacity, while also increasing failure toughness and permeability and mitigating crack propagation and swell-shrink tendencies^16,17^. While substantial research has been devoted to the static properties of fiber-reinforced soil, the dynamic properties have garnered less attention. Previous investigations have employed dynamic triaxial and resonance column tests to assess the influence of various parameters on the dynamic stiffness, strength, and damping ratio of fiber-reinforced soil. For instance, Maher et al.^18^ conducted dynamic triaxial tests on fiber-reinforced sand, demonstrating a notable enhancement in both dynamic modulus and damping ratio for medium-grain sand. Similarly, Jamshidi et al.^19^ utilized shaking table tests on fiber-reinforced fine sand and observed a significant improvement in its dynamic stiffness and strength. Various scholars have identified key factors affecting the dynamic characteristics of fiber-reinforced soil, including the fiber addition rate, consolidation confining pressure, and loading–unloading cycles^20,21^. Pradhan et al.^22^ proposed the notion of "optimal content" after finding that dynamic stiffness and strength initially increased with fiber content. Amir and Aggour^23^ noted that the damping ratio was positively correlated with fiber content, irrespective of the optimal addition rate. Sadeghi and Beigi^20^ highlighted that while the dynamic shear modulus decreases with the increase in deviator stress ratio, the rate of loss for fiber-reinforced soil is considerably lower compared to plain soil. Research in this domain within China has been underway for over a decade.

The existing literature predominantly focuses on the effects of polypropylene fibers on clay and sand substrates, leaving a research gap in the dynamic properties of silt soil reinforced with recycled polyester fibers. To address this lacuna, the present study employs regenerated polymer fibers and inorganic curing agents to enhance the structural integrity of silty soil subgrades, subsequently analyzing both the dynamic characteristics and microscopic reinforcement mechanisms involved. Our findings reveal that fiber incorporation mitigates inclined cracking in the enhanced soil, leading to ductile failure modes. The primary reinforcement effects manifest in the augmentation of soil confining pressure, thereby boosting the overall strength and dynamic characteristics of the fiber-reinforced soil. Moreover, the fiber addition improves the soil's tensile capacity, effectively curtailing crack propagation. Concurrently, fibers facilitate the interconnection of smaller soil particles to form aggregates, yielding a more compact and stable soil matrix. These research outcomes not only contribute to the optimization of silt soil subgrade reinforcement techniques but also support the sustainable reuse of waste polyester bottles, reducing environmental pollution stemming from improper disposal.

## Test materials and methods

### Test materials

The test soil was sourced from a highway located in Yancheng, Jiangsu Province. Table 1 presents the soil's fundamental physical properties. The soil sample has a liquid limit water content ${\text{W}}_{\text{L}}$ < 50% and a plasticity index ${\text{I}}_{\text{P}}$ < 10, classifying it as a low liquid limit silt soil according to the *soil test specification* (SL 237-1999) in China. The Renewable Polyester Fiber, procured from a textile factory in Tai'an, exhibits a density ranging between 1.31 and 1.37 g/cm^3^, tensile strength (for bundle monofilament) between 200 and 400 MPa, and an elongation at break spanning 140.6–154.7%. Table 2 outlines the essential physical attributes of quicklime, while Table 3 provides a chemical composition analysis, indicating the lime as calcarious grade III quicklime, suitable for creating alkaline conditions. Notably, when compared to cement, fly ash proves to be a more cost-effective option, and the freeze–thaw cycle capability of fly ash reinforced soil surpasses that of cement. Table 4 details the primary properties of fly ash. The gypsum utilized in this research is recycled building gypsum powder, with calcium sulfate hemihydrate (CaSO_4_·1/2H_2_O) as its primary constituent.

**Table 1:** Physical indexes of silt.

Natural moisture content (%)	Liquid limit (%)	Plastic limit (%)	The plastic index	Particle size distribution (%)			Maximum dry density (g/cm^3^)	Optimal moisture content (%)	Proportion (g/cm^3^)	pH
				< 5 μm	5-75 μm	> 75 μm				
25.4	31.6	22.8	8.8	11.3	79.8	8.9	1.81	16.45	2.71	8.21

**Table 2:** Basic physical indexes of quicklime.

Proportion	pH	Clay content (%) (< 2 μm)	Silt content (%) (2–75 μm)	Sand content (%) (> 75 μm)
3.31	12.4	5.4	42.7	51.9

**Table 3:** Chemical composition analysis of quicklime.

Chemical composition	CaO	SiO_2_	Al_2_O_3_	Fe_2_O_3_	MgO	SO_3_	Na_2_O	K_2_O	TiO_2_	SrO	MnO	Ignition loss
Content (%)	65.23	2.62	1.16	0.74	0.46	0.13	0.20	0.18	0.053	0.029	0.028	24.36

**Table 4:** Main index of fly ash.

Main chemical composition (%)				Proportion	Optimum moisture content (%)	Maximum dry density (g/cm^3^)
CaO	Fe_2_O_3_	Al_2_O_3_	SiO_2_			
2.8	7.9	28.4	46.2	2.15	23.2	1.34

### Test scheme

Tang et al.^24^ demonstrated that both lime and fly ash contribute to enhancing the curing compressive strength of soil. Specifically, lime acts as an activator for the latent hydraulic properties of fly ash, while fly ash contributes to stabilizing the lime structure. Nevertheless, an excess of either component diminishes the effectiveness in boosting compressive strength. For optimal results, the proportion of these materials can be finely tuned^25–27^. Lin et al.^28^ established that a 1:2 ratio of quicklime to fly ash yields the most effective consolidation of soft soil. Karim et al.^29^ noted that soils reinforced with lime and fly ash exhibit low early strength. To address this, the present study adopts the soft soil reinforcement strategy proposed by Lin et al.^28^, incorporating 3% building gypsum powder (CaSO_4_·1/2H_2_O) as an admixture to enhance the early strength of soil samples. Gypsum powder facilitates early solidification and synergistically acts with lime and fly ash to improve soil strength. Upon exposure to water, the building gypsum powder undergoes rehydration to form dihydrate gypsum, resulting in a hard, stone-like material. The corresponding chemical reaction is: CaSO_4_·1/2H_2_O + 3/2H_2_O = CaSO_4_·2H_2_O.

In alignment with the findings of Chaduvula et al.^30^, the present study incorporates a range of fiber content levels, denoted as mass ratios (fiber mass to total sample mass), including 0, 0.1%, 0.2%, 0.3%, 0.5%, and 0.7%. The lengths of the fibers are specified at 6 mm, 9 mm, 12 mm, 15 mm, and 17 mm. The mass ratios for the quicklime and fly ash admixtures, in relation to the total sample mass, are set at 4% and 8%, respectively. Table 4 elaborates on the experimental design, detailing the physical and mechanical properties of the enhanced soil samples. It should be noted that, aside from the tests concerning boundary moisture content and particle size, soil samples are adjusted to their optimal water content.

### Test methods

#### Dynamic triaxial test

**Figure 1:**
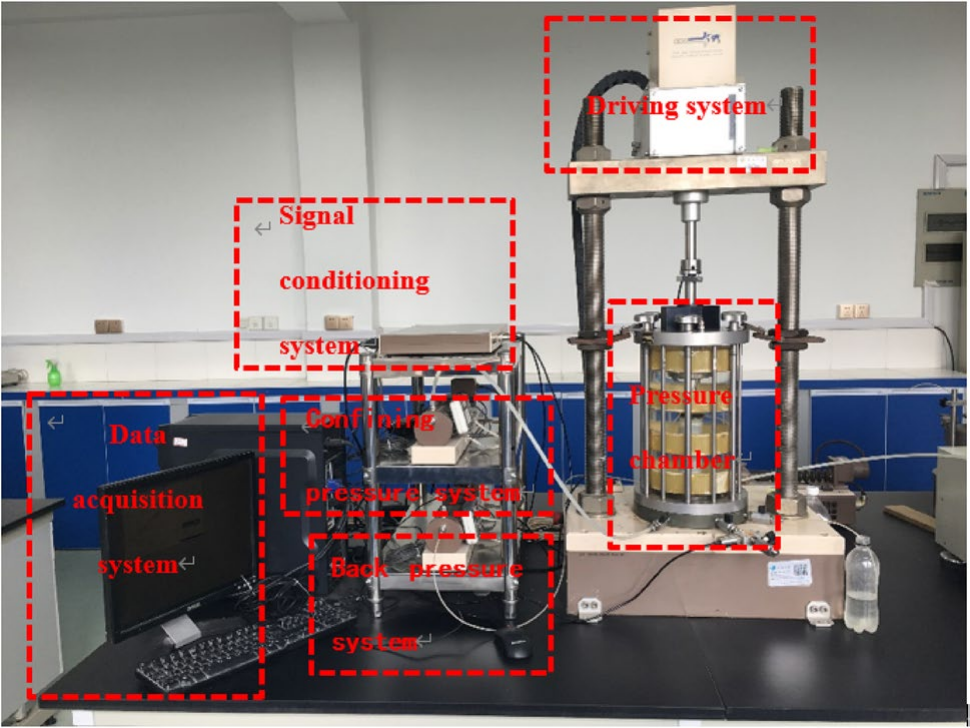
GDS dynamic triaxial apparatus.

The dynamic triaxial test was conducted using the GDS dynamic triaxial apparatus provided by Southeast University (model: DYTTS, manufacturer: GDS Instrument and Equipment Co., Ltd. in the UK), as shown in Fig. 1.This instrument encompasses various components, including a pressure chamber, driving system, confining pressure control system, back pressure control system, data acquisition system, and signal conditioning system. Employing a stress-controlled loading approach, the test conditions were as follows: a confining pressure of 20 kPa and a back pressure of 10 kPa were applied for head saturation with a duration of 24 h. For back pressure saturation, the confining pressure was set at 100 kPa, the back pressure at 90 kPa, and the saturation time extended to 24 h, achieving a B value exceeding 0.95.

In accordance with the research findings of Cai et al.^31^ regarding the dynamic characteristics of soft clay under vehicular load, a sinusoidal wave was utilized as the input form for dynamic load. The experiment was set at a vibration frequency of 1 Hz, with effective confining pressures spanning 20 kPa, 40 kPa, 60 kPa, 80 kPa, and 100 kPa. The consolidation ratio ${\text{K}} \, = \, {\sigma}_{1}/{\sigma}_{3}$ was maintained at 1.0. Dynamic stress amplitudes were defined using the dynamic shear stress ratio ${\text{S}}\, = { \sigma}_{\text{d}}/{{2}{\sigma}}_{3}$, which took values of 0.05, 0.1, 0.15, and 0.25. The dimensions of the soil samples were specifically 39.1 mm in diameter and 80 mm in height.

#### SEM test

The qualitative examination of pore alterations, the development of cementitious materials, and microstructural transformations before and after the incorporation of admixtures provides valuable insights into the internal mechanisms responsible for silt soil improvement. Subsequent to a 28-day curing period, marked enhancements were observed in the durability, strength, and roadworthiness of the modified silt soil. Electron microscopy analyses were performed at both 7-day and 28-day intervals to compare and evaluate pore-filling and particle-bonding characteristics between the untreated and improved soil samples.

The test samples were fabricated using the static pressure method, yielding both modified silty soil and native silty soil samples, each with dimensions of 50 mm in diameter and 100 mm in height. Following sealing, these specimens were stored in a standard curing chamber for 28 days to meet requisite curing conditions. Subsequently, a fresh cross-section, approximately 1 cm^2^ in size, was excised from each sample. These sections were then flash-frozen and subjected to scanning electron microscopy (SEM) analysis.

## Results and analysis

### Dynamic characteristics

The dynamic constitutive relationship of soil, encapsulating the stress–strain relationship, serves as a vital reference for analyzing soil's dynamic instability processes and for calculating its finite element dynamic properties^32^. Under dynamic loading conditions, this relationship often manifests nonlinear and hysteretic behaviors. Factors such as soil anisotropy, variations in stress pathways, and energy dissipation during shear deformation significantly influence the precise determination of the constitutive relationship. For minor dynamic loads, elastic deformation predominates, while plastic deformation becomes increasingly significant with escalating dynamic load magnitudes. During high-amplitude dynamic loading events, such as strong earthquakes or blast-induced stresses, marked changes occur in the soil's structural composition. Therefore, understanding the intrinsic relationship between dynamic stress and dynamic strain is crucial for assessing soil strength and deformability under dynamic loading, as well as for predicting vibrational and liquefactive failure in sandy and silty soils^2^.

**Figure 2:**
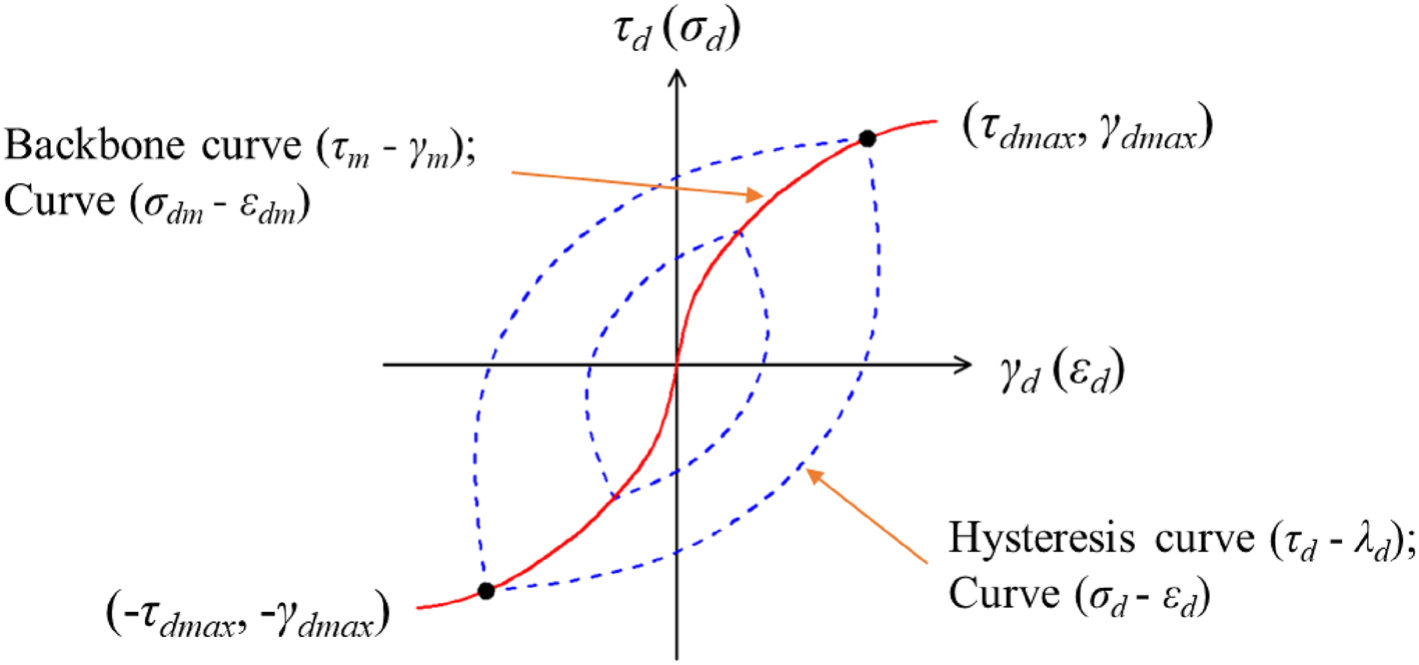
Dynamic stress—dynamic strain hysteresis curve and backbone curve.

In cyclic reciprocating shear tests, stress is applied along a plane where the initial shear stress of the soil mass is zero. The resulting stress–strain relationship during a loading cycle forms a hysteresis loop. The vertices of this loop, representing the maximum periodic shear strain (${\pm}{\gamma}_{\text{max}}$) and the maximum periodic shear stress (${\pm}{\tau}_{\text{max}}$) under varying dynamic stress conditions, delineate what is termed the stress–strain backbone curve of the soil^33^. Figure 2 illustrates both the dynamic stress-dynamic strain hysteresis curve and the backbone curve. The backbone curve characterizes the nonlinear dynamic strain, indicating the developmental trend between maximum shear stress and maximum shear strain. Conversely, the hysteresis curve reveals the time-dependent relationship between shear stress and shear strain within a single stress cycle, thereby encapsulating the overall dynamics of the stress–strain relationship^22^.

During the dynamic triaxial test, axial dynamic stress (${\sigma}_{\text{d}}$) is applied, and the corresponding axial dynamic strain (${\varepsilon}_{\text{d}}$) is measured. This enables the plotting of a hysteresis curve for each cycle. By varying the dynamic stresses (${\sigma}_{\text{d}}$), a backbone curve can be constructed based on the maximum values of dynamic stress (${\sigma}_{\text{dmax}}$) and dynamic strain (${\varepsilon}_{\text{dmax}}$). Additionally, the maximum dynamic shear strain (${\gamma}_{\text{dmax}}$) can be derived from the measured axial dynamic strain (${\varepsilon}_{\text{d}}$). Using the obtained dynamic elastic modulus (${\text{E}}_{\text{d}}$), the dynamic shear modulus (${\text{G}}_{\text{d}}$) can be calculated through a specific conversion relationship.

1 $$\left\{\begin{array}{l}{\gamma}_{\text{d}} \, {=} \, {\varepsilon }_{d}\text{(}{1} \, {+} \, {\mu}\text{)} \, \, \, \, \, \, \\ {\text{G}}_{\text{d}} \, {=} \, {\text{E}}_{\text{d}} \, {/} \, {2}\text{(}{1} \, {+} \, {\mu}\text{)}\end{array}\right.$$

Consequently, it can be inferred that the relationships between dynamic stress and dynamic strain, as well as between shear stress and shear strain, follow similar principles. Analysis of test data reveals that certain models closely approximate these relationships, with the viscoelastic model demonstrating the greatest similarity. Therefore, researchers have prioritized the study of viscoelastic models in the development of dynamic constitutive models for soil. Additionally, elastic–plastic models are experiencing rapid advancements. These models address the dynamic stress–strain characteristics of soil from various perspectives, specifically in terms of nonlinearity (represented by the backbone curve) and hysteresis (represented by the hysteresis curve).

**Figure 3:**
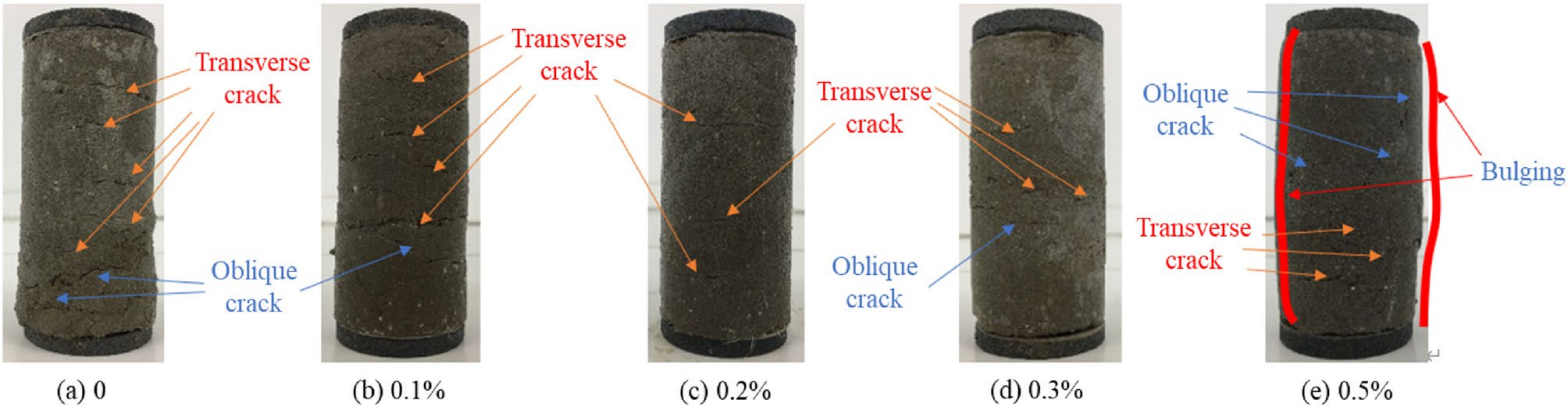
Failure modes of improved soil with different fiber content.

**Figure 4:**
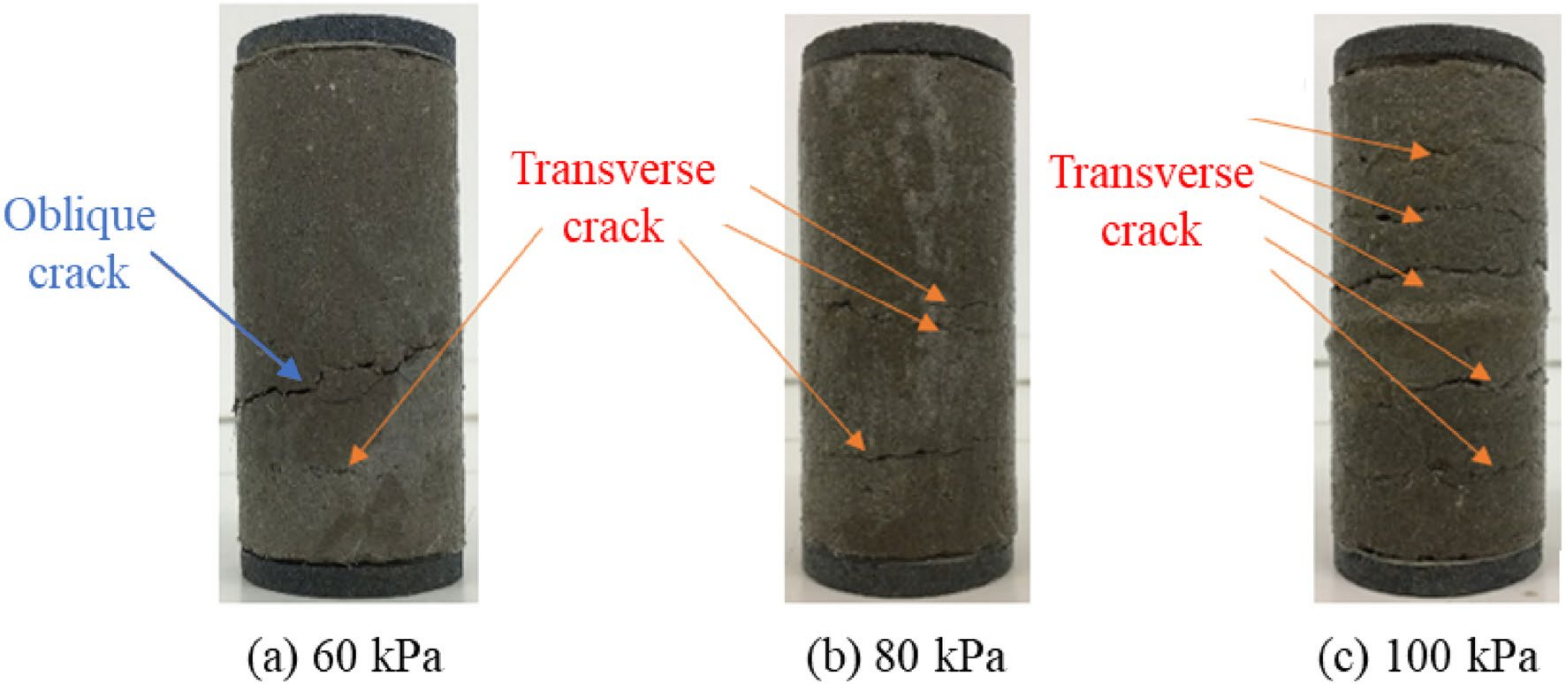
Failure modes of improved soil under different confining pressures.

Figure 3 illustrates the failure characteristics of soil improved with varying fiber content under a confining pressure of 80 kPa. Figure 4 presents the failure modes observed in the improved soil with a fiber content of 0.2% under different confining pressures (60 kPa, 80 kPa, 100 kPa). In the case of plain modified soil (absent of fiber), the failure characteristics are marked by conspicuous expansion of transverse cracks and the presence of inclined cracks, suggesting a level of brittle failure. As the fiber content increases, the frequency of inclined cracks diminishes, signifying enhanced sample toughness and a shift toward ductile failure. Specifically, at a fiber content of 0.2%, the soil sample exhibits the least amount of damage, indicating optimal resistance to failure.

The dynamic stress–strain curve for improved silty soil exhibits pronounced nonlinearity, akin to that observed in generic improved soil. The curve closely approximates a hyperbolic shape^34^. The mathematical expression for this relationship is as follows:

2 $${\sigma}_{\text{d}} \, {=} \, \frac{{\varepsilon}_{\text{d}}}{{\text{a}} \, {+} \, {\text{b}}{\varepsilon}_{\text{d}}}$$

where ${\sigma}_{\text{d}}$ is the dynamic stress; ${\varepsilon}_{\text{d}}$ is dynamic strain; a and b are constants.

**Figure 5:**
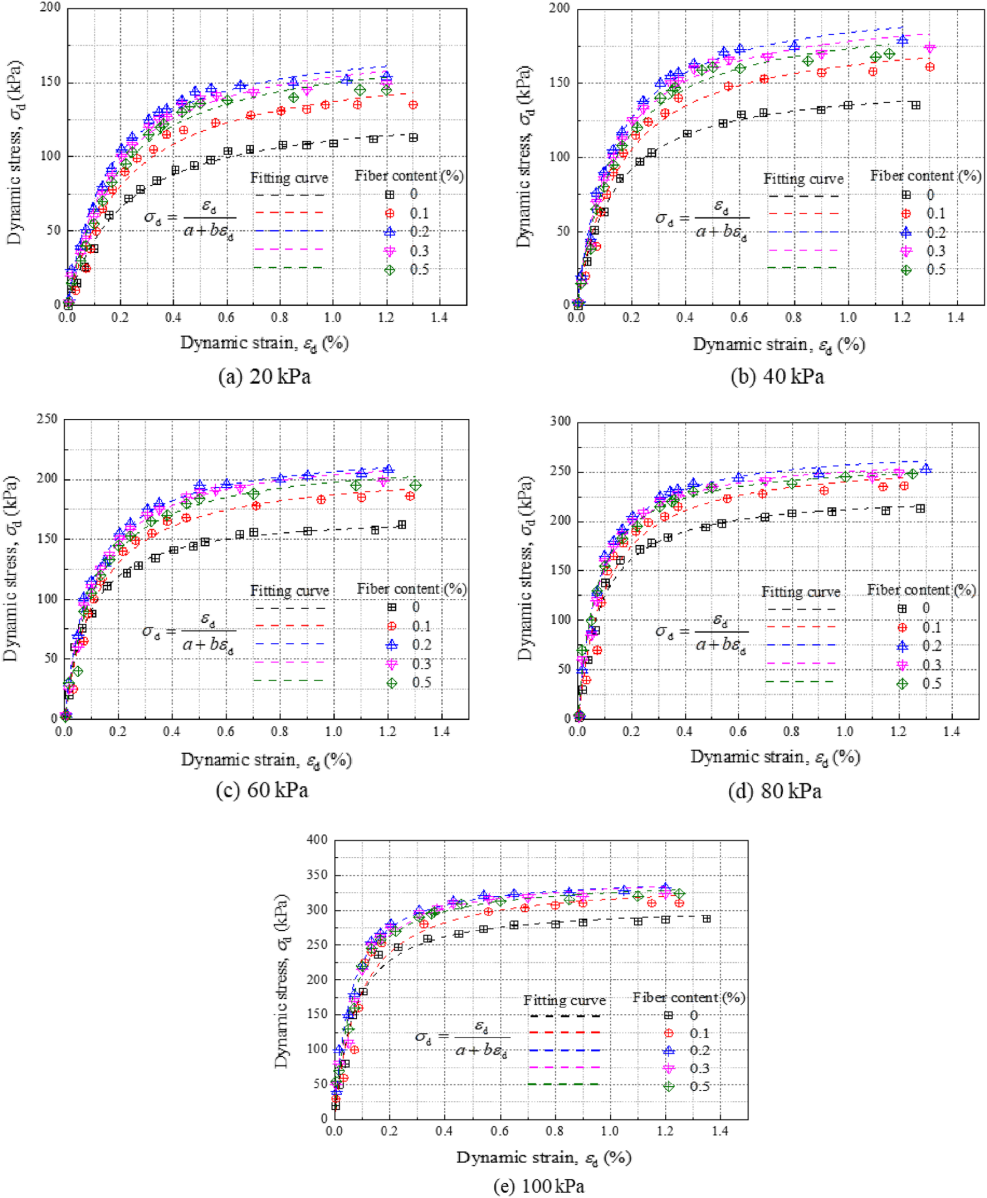
Dynamic stress–strain relation curves of modified silt under different confining pressures.

Equation 2 is employed to analyze the dynamic stress–strain behavior of enhanced silty soil samples subjected to varying confining pressures; the corresponding results are depicted in Fig. 5. For dynamic strains below 0.1%, the stress–strain curves for the soil fortified with different fiber concentrations are virtually indistinguishable. However, for strains exceeding 0.2%, these curves diverge and ultimately stabilize. Compared to the unmodified soil, the soil amended with fiber exhibits a substantial increase in dynamic load-bearing capacity. Additionally, as the confining pressure escalates, the dynamic strength of the modified soil also experiences a commensurate increase. Moreover, when both the dynamic strain level and fiber content are set at 0.2%, higher amplitudes of dynamic stress result in enhanced cyclic load-bearing capabilities.

Compared to both the dynamic strength of pure soil and that of modified plain soil, the incorporation of fiber markedly enhances the soil's resilience to dynamic loading. This dynamic strength demonstrates a positive correlation with increasing pressure. Notably, when the fiber content is calibrated to 0.2%, the load-bearing capability of the modified plain soil reaches its peak under identical dynamic loading conditions.

The ${\text{E}}_{\text{d}}-{\varepsilon}_{\text{d}}$ relation is further obtained from the ${\sigma }_{\text{d}} - {\varepsilon}_{\text{d}}$ relation curve:

3 $${\text{E}}_{\text{d}} \, \text{=} \, {1} \, {/} \, \text{(}{\text{c}} \, {+} \, {\text{d}}{\varepsilon}_{\text{d}}\text{)}$$

where ${\sigma}_{\text{d}}$ is the dynamic stress; ${\varepsilon}_{\text{d}}$ is dynamic strain; c and d are constants.

**Figure 6:**
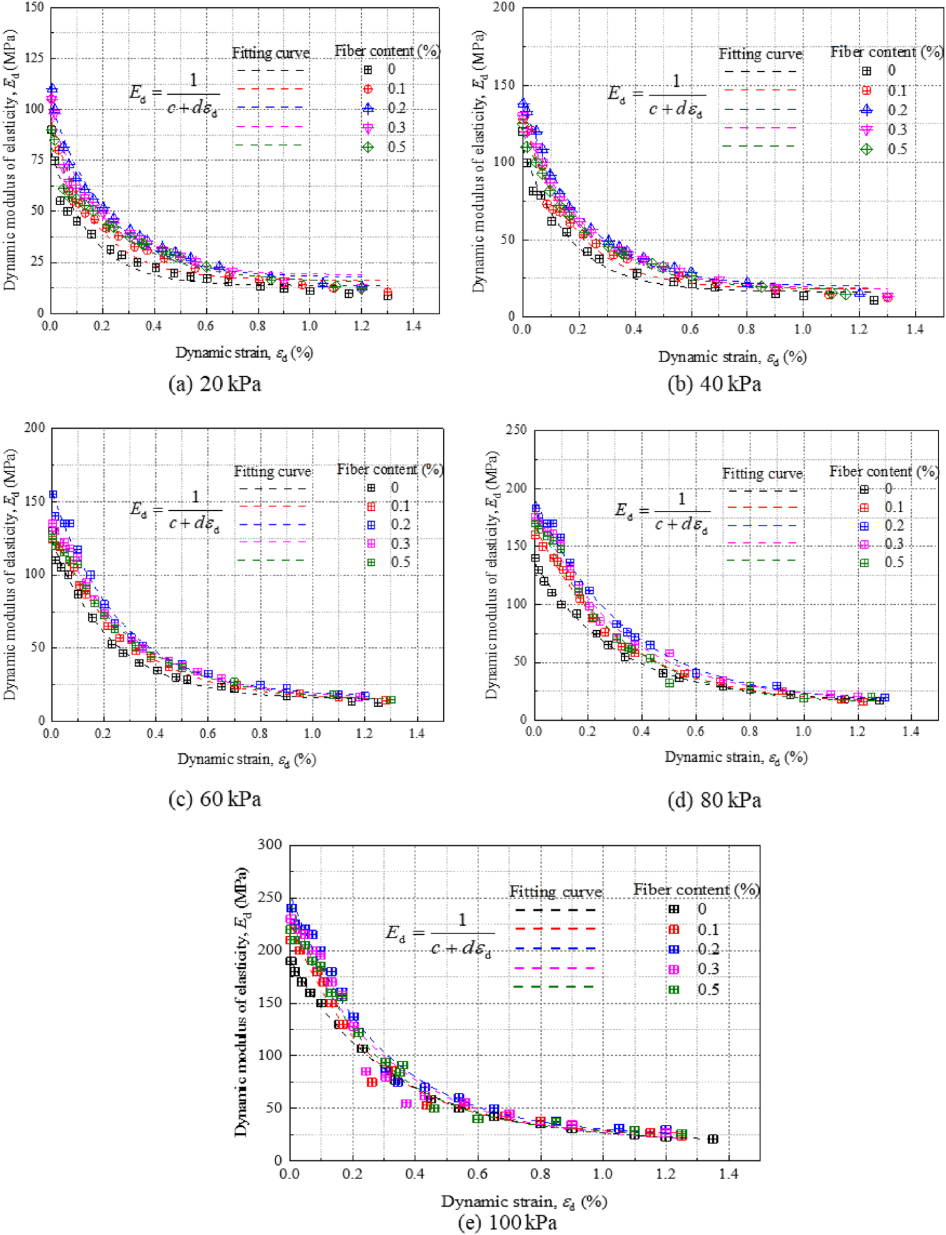
Dynamic modulus-dynamic strain relation curve of modified silt under different confining pressures.

Equation (3) is employed to fit the relationship between dynamic elastic modulus and dynamic strain for enhanced silty soil samples under varying confining pressures. The fitted outcomes are depicted in Fig. 6. As dynamic strain evolves, the dynamic elastic modulus, represented by ${\text{E}}_{\text{d}} - \, {\varepsilon}_{\text{d}}$, consistently diminishes under five different confining pressures. This trend aligns with findings from cement-enhanced silty soils^35^, lime-amended subgrade soils^36^, and fiber-enhanced fly ash soils^37^. The modulus converges to a stable value as dynamic strain increases. Within a dynamic strain range of 0.002, the curve is notably steep; the modulus decreases precipitously as strain increases. However, for strains exceeding 0.004, the rate of decrease moderates and the curve levels off. Under identical conditions of confining pressure and dynamic strain, soil modified with 0.2% fiber content exhibits the highest dynamic elastic modulus. As confining pressure escalates, the curves converge, signifying that fiber content exerts a minor influence compared to the substantial impact of confining pressure. This increased pressure constrains the transverse deformation of the modified soil, which manifests as an augmented dynamic elastic modulus. The cohesiveness between the improved soil and fiber intensifies due to hydration and pozzolanic reactions, thereby bolstering the soil's resistance to deformation^38^.

### Quasi-cohesive force reinforcement effect

The concept of quasi-cohesion is employed to articulate the strength attributes of fiber-reinforced soil, as expressed in the following equation:

4 $$\left\{\begin{array}{l}{\sigma}_{1} \, {=} \, {\sigma}_{3}{{\text{tan}}}^{2}{(45^{ \circ} +}{\varphi}{/2)} \, {+} \, {2}{\text{c}}{\text{tan}}^{2}{(45 ^{\circ} +}{\varphi}{/2)} \, {\text{F}}{\text{i}}{\text{b}}{\text{e}}{\text{r}} \, {\text{s}}{\text{a}}{\text{n}}{\text{d}} \, \, \, \, \, \, \, \, \, \\ {\sigma}_{1} \, {=} \, {\sigma}_{3}{{\text{tan}}}^{2}{(45 ^{\circ}+}{\varphi}{/2)} \, {+} \, \text{2(}{\text{c}}{+} \Delta \text{c}\text{)}{\text{tan}}^{2}{(45^{ \circ}+}{\varphi}{/2)} \, {\text{F}}{\text{i}}{\text{b}}{\text{e}}{\text{r}} \, {\text{c}}{\text{l}}{\text{a}}{\text{y}}\end{array}\right.$$

Let ${\sigma}_{3}$ represent the confining pressure applied to clay soil, and ${\sigma}_{1}$ symbolize the major principal stress when the soil reaches limit equilibrium. Under these conditions, the Mohr circle characterized by principal stresses ${\sigma}_{1}$ and ${\sigma}_{3}$ is tangent to strength line 1. When fibrous clay is subjected to a confining pressure of ${\sigma}_{3}$ but has not yet attained limit equilibrium or a minor principal stress of ${\sigma}_{3}$ , the value of ${\sigma}_{1}$ must be increased to ${\sigma}_{{1}{\text{f}}}$ for equilibrium to be achieved. Consequently, the confining pressure becomes ${\sigma}_{{3}{\text{f}}}$, and the Mohr circle is now tangent to strength line 1 with principal stresses ${\sigma}_{{1}{\text{f}}}$ and ${\sigma}_{{3}{\text{f}}}$. To attain a confining pressure of ${\sigma}_{3}$ in fiber-reinforced clay, the major principal stress must reach ${\sigma}_{{1}{\text{f}}}$ .In this case, the Mohr circle, defined by ${\sigma}_{{1}{\text{f}}}$ and ${\sigma}_{3}$, is tangent to strength line 2. Significantly, ${\sigma}_{1}{{(\delta}{\sigma}}_{1} \, {>} \, {0)}$, indicating an enhancement in the soil's strength, thereby underscoring the efficacy of fiber reinforcement.

In sandy soils, cohesion is absent prior to fiber reinforcement; however, post-reinforcement, a form of cohesion emerges, commonly termed "quasi-cohesion" or "apparent cohesion" in existing literature^39^. During triaxial testing, reinforced sandy soil is typically treated as analogous to unreinforced sandy soil. To achieve equilibrium in fiber-reinforced sandy soil, certain criteria must be satisfied:

5 $$\left\{\begin{array}{l}{\sigma}_{{1}{\text{f}}} \, {=} \, \text{(}{\sigma}_{3}{+}{\delta}{\sigma}_{3}\text{)}{\text{tan}}^{2} {(45^{\circ} +}{\varphi}{/2)} \, \, \, \, \, \, \, \, \, \, \, \, \, \, \, \, \, \, \, \, \, \, \, \, \, \, \, \, \, \, \, \, \, \, \, \, \, \, \, \, \, \, \, \, \, \, \, \, \, \, \, \\ {\sigma}_{{1}{\text{f}}} \, {=} \, {\sigma}_{3}{{\text{tan}}}^{2} {(45^{\circ}+}{\varphi}{/2)} \, {+} \, {\Delta}{\sigma}_{3}{{\text{tan}}}^{2} {(45^{\circ} +}{\varphi}{/2)} \, \, \, \, \, \, \, \, \, \, \, \, \, \, \, \, \, \, \, \, \, \, \, \, \, \, \\ {\sigma}_{{1}{\text{f}}} \, {=} \, {\sigma}_{3}{{\text{tan}}}^{2} {(45^{\circ} +}{\varphi}{/2)} \, {+} \, \text{[}{\Delta}{\sigma}_{3}{{\text{tan}}}^{2}{(45^{\circ}+}{\varphi}{/2)}\text{]}{\text{tan}}{(45^{\circ}+}{\varphi}{/2)}\end{array}\right.$$

Among them, the parameter *c* in Eq. (4) meets the following condition:

6 $${2}{\text{c}}{ = \Delta}{\sigma}_{3}{\text{tan}}{(45 ^{\circ} +}{\varphi}{/2)}$$

Consequently, it is evident that the quasi-cohesion observed in fiber-reinforced sandy soil is induced by an incremental change in the confining pressure, denoted as ${\Delta}{\sigma}_{3}$. This is akin to an initial confining pressure ${\sigma}_{3}$ in the fiber-reinforced sandy soil that subsequently escalates to ${\sigma}_{3} \, {+} \, {\Delta}{\sigma}_{3}$. Such an increase results in enhanced strength and improved dynamic properties of the fiber-reinforced sandy soil, aligning closely with the strength mechanisms observed in fiber-reinforced clay soils. Nevertheless, as the increment in confining pressure due to fiber reinforcement is not directly measurable, scholars concur that both ${\Delta}{\sigma}_{3}$ and quasi-cohesion can be quantified through the following equation:

7 $$\left\{\begin{array}{l}{\Delta}{\sigma}_{3} \, {/} \, {\sigma}_{3} \, {=} \, {\Delta}{\sigma}_{{1}{\text{f}}} \, {/} \, {\sigma}_{1} \, \, \, \, \, \, \, \, \, \\ {\text{c}} \, {=} \, \text{[}{\sigma}_{3}\text{(}{\Delta}{\sigma}_{{1}{\text{f}}} \, {/} \, {\sigma}_{1}\text{)}{\text{k}}_{\text{p}}^{1/2}\text{]} \, {/} \, {2}\end{array}\right.$$

### Interfacial friction of reinforced soil

**Figure 7:**
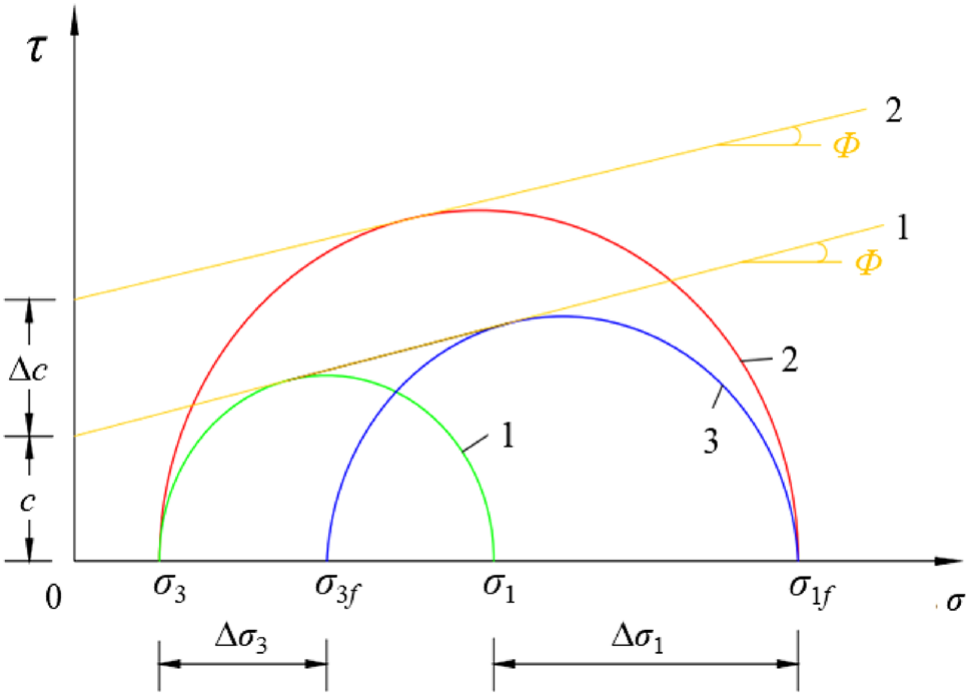
Schematic diagram of stress circle of fiber-reinforced and unreinforced clay.

**Figure 8:**
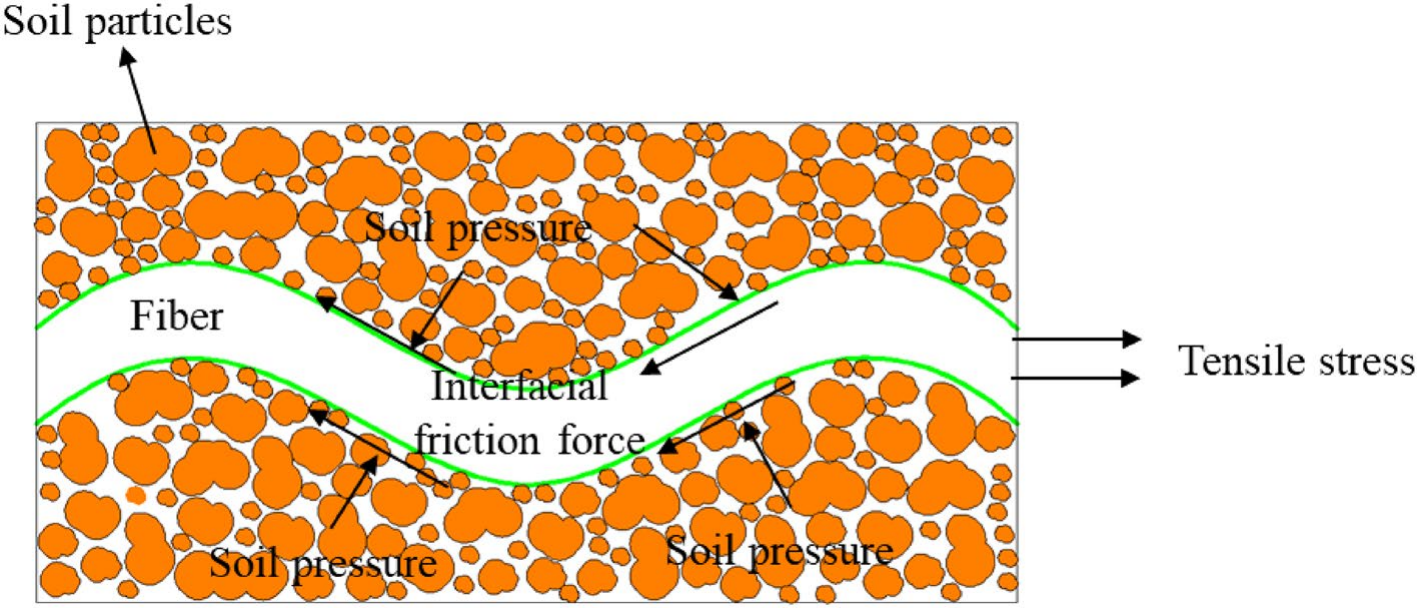
Soil particle and fiber friction effect.

Tang et al.^40^ identified three primary forces exerted by fiber-reinforced soil on the fibers: (1) Interfacial friction between the fibers and soil particles, as depicted in Fig. 7. This friction arises when fibers, subjected to tensile stress within the soil, are compressed by adjacent soil particles; (2) Barrier force between fibers and soil particles, illustrated in Fig. 8. Under extrusion forces, some soil particles embed themselves into the fiber surface, enabling the fibers to resist the movement of these particles through tensile action; (3) Matric suction, also shown in Fig. 8. In an unsaturated state, matric suction occurs at the water–air interface, enhancing soil mass strength and playing a crucial role in geotechnical engineering applications such as slope stabilization and foundation pit support^40^. Furthermore, this generated matric suction impedes fiber movement.

When the fiber content is low, the fibers are uniformly distributed within the soil, enabling comprehensive contact with the soil particles. Upon application of an external load during testing, the resultant tensile stress in the soil triggers both friction and adhesion between the soil particles and fibers. Conversely, Chaduvula et al.^30^ indicated that elevated fiber content may result in fibers intertwining within the soil, leading to clumping. Such agglomeration can subsequently induce the formation of weaker zones around the fiber clumps, thereby constraining any enhancement in the soil's strength due to fiber reinforcement.

**Figure 9:**
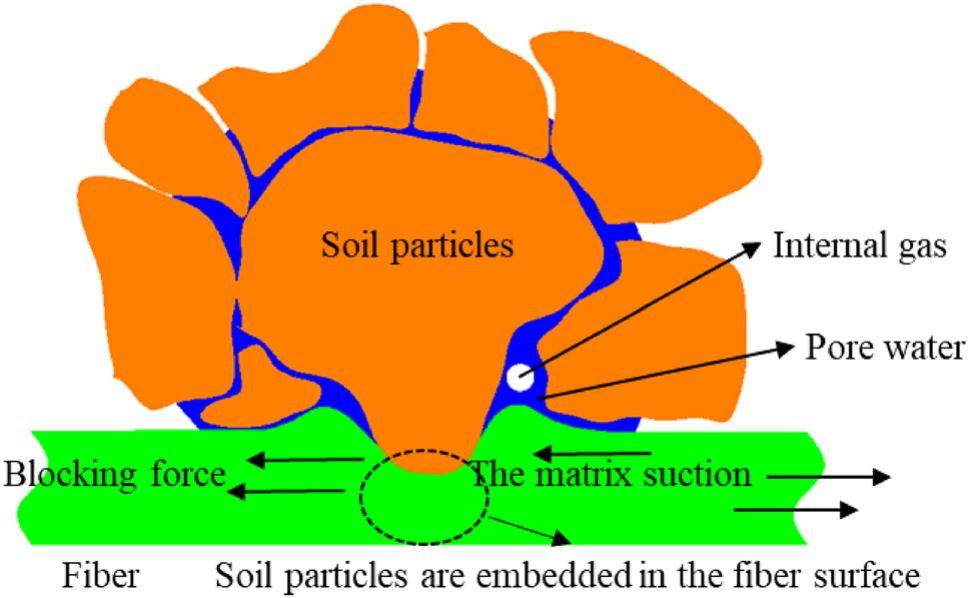
Soil particles embedded effect.

When fiber-reinforced soil reaches its tensile strength limit, it manifests cracking patterns. During this phase, fibers present on the soil's failure surface act as connective "bridges" rather than undergoing pull-out or rupture. Consequently, the soil remains in a state of "cracking but not breaking," thereby sustaining additional tensile stresses from external loads. This state contributes to substantial residual tensile strength and effectively curtails further crack propagation^41^, as depicted in Fig. 9. The efficacy of this "bridging" mechanism is directly proportional to the density of fibers on the failure surface: higher fiber content markedly enhances residual tensile strength. However, if the applied tensile stress exceeds either the tensile strength of the fibers or the shear strength at the soil-reinforcement interface, the fibers may be extracted or fractured, nullifying their reinforcing effect^41^.

**Figure 10:**
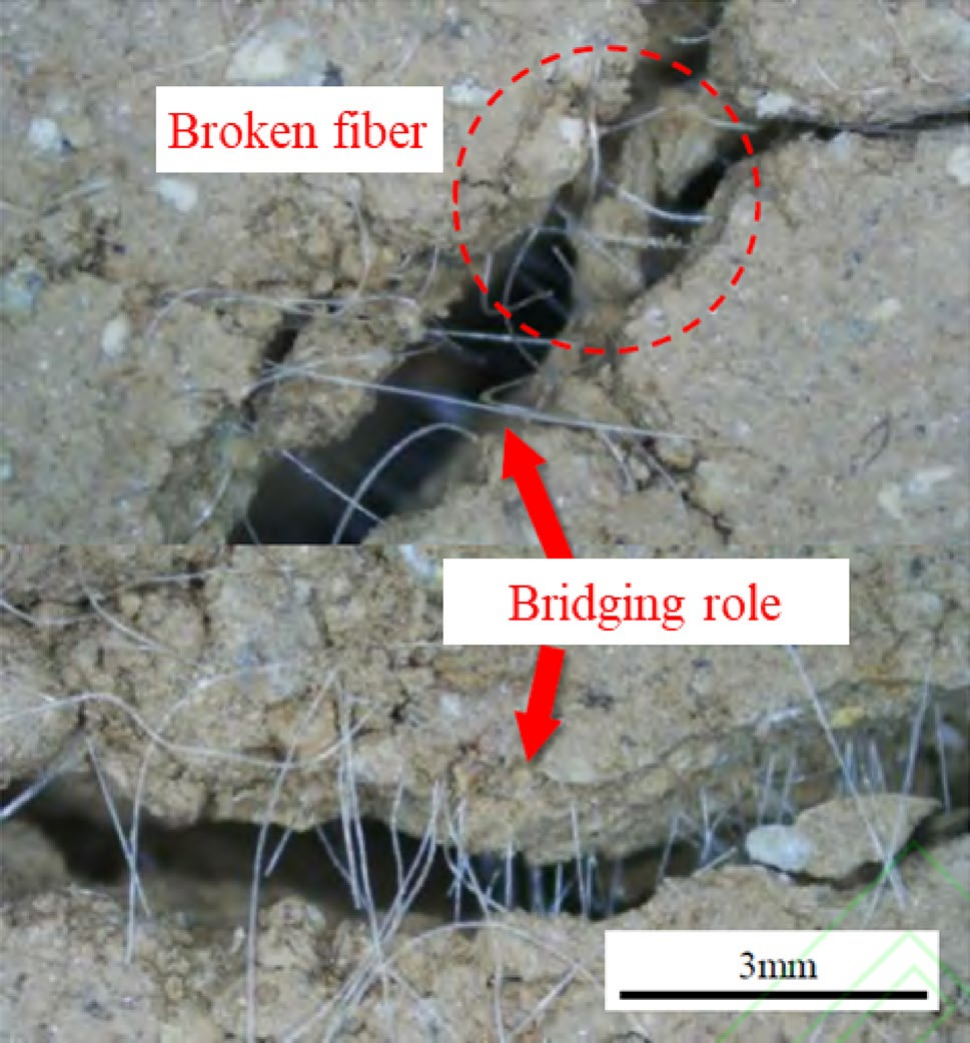
"Bridge" reinforcement effect of fiber in fiber soil.

From a microscopic vantage point, Fig. 10 presents a Scanning Electron Microscope (SEM) micrograph illustrating the interface between soil particles and recycled polyester fibers. The SEM images reveal an uneven topography on the fiber surface characterized by folds and pits^42–45^. This surface morphology enhances the adherence or embedment of soil particles onto the fiber. When subjected to external forces, the disparate elastic moduli of the fibers and soil particles result in varied deformation and displacement^46^. This discrepancy amplifies the interlocking forces and friction at the soil-fiber interface, thus redistributing a portion of the external load and enhancing the integrity of the fiber-soil matrix.

**Figure 11:**
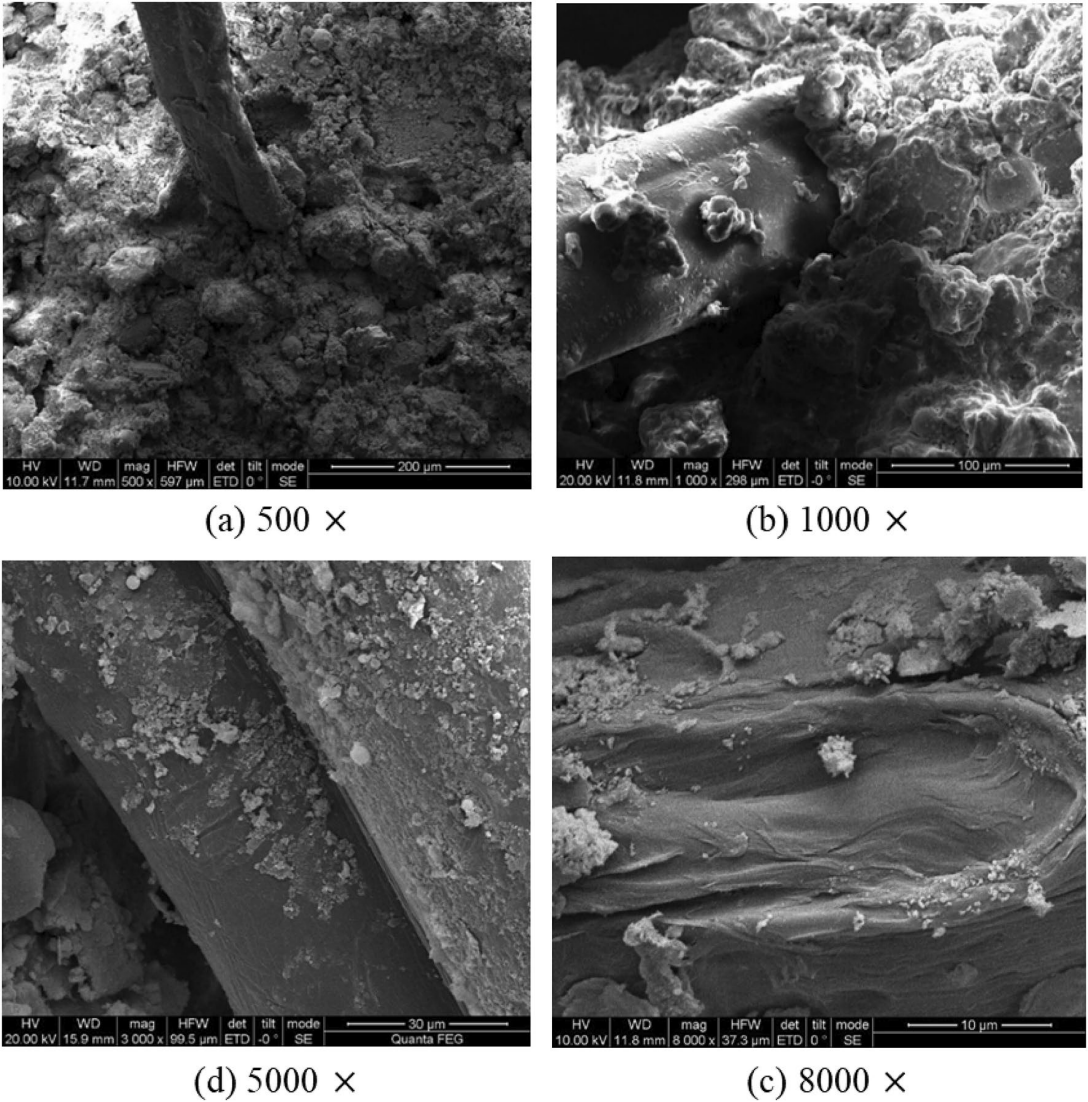
Micrograph of interface between soil particle and fiber.

**Figure 12:**
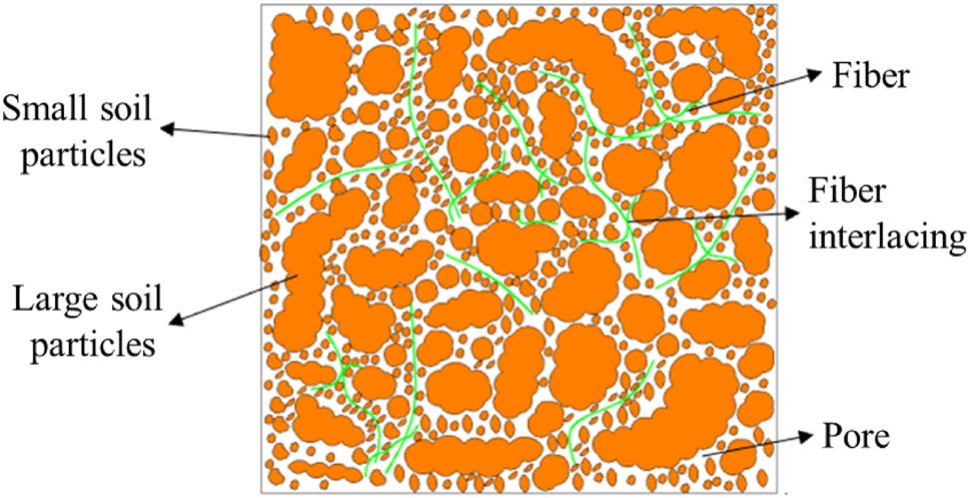
Schematic diagram of fiber distribution in soil particles.

The incorporation of recycled polyester fibers in the soil matrix results in a randomly distributed, three-dimensional network structure, as illustrated in Fig. 11. This architecture gives rise to an "interleave" effect^47,48^. Within this structure, individual fibers not only sustain forces but also exert tensile forces on adjacent fibers. Consequently, mechanical loads are more uniformly distributed throughout the soil mass (see Fig. 12), enhancing the spatial constraint effect on the fiber-reinforced soil and rendering it more stable under dynamic conditions.

## Conclusion

In this study, comprehensive analyses were conducted through triaxial tests and Scanning Electron Microscopy (SEM) on silty soil enhanced with recycled polyester fibers. The objective was to elucidate the impact of varying confining pressure conditions and fiber content on the soil's physical and mechanical properties. The key findings are summarized as follows:

1. When compared to unmodified improved soil, an increase in fiber content contributes to a reduction in inclined cracks within the improved soil, progressively evolving towards ductile failure. Particularly, when the fiber content is 0.2%, the fiber-modified soil sample demonstrates the least degree of damage, supreme dynamic strength and elastic modulus, and optimal resistance to dynamic loads.
2. The enhanced cohesion in fiber-reinforced soil, termed as "quasi-cohesion," arises from an increment in confining pressure, denoted as ${\Delta}{\sigma}_{3}$. This is functionally equivalent to an initial confining pressure ${\sigma }_{3}$ in fiber-reinforced soil being increased to ${\sigma}_{3}{+\Delta}{\sigma}_{3}$. Consequently, both the strength and dynamic properties of the fiber-reinforced soil are improved.
3. Upon reaching its strength limit and subsequent crack appearance, fiber-reinforced soil sustains a "cracked but continuous" state due to the intervening fibers, thereby manifesting notable residual tensile strength and markedly inhibiting further crack progression. Furthermore, cementitious materials envelop particles within the enhanced soil structure, facilitating the amalgamation of smaller soil particles that subsequently occupy interstitial spaces, culminating in a denser and more stable soil structure. This methodology posits applicative value in the realms of coastal city construction and foundation reinforcement.

## Data Availability

The datasets used and analysed during the current study available from the corresponding author on reasonable request.
